# Supplementary figures and images for: A contrast-enhanced CT-based whole-spleen radiomics signature for early prediction of oxaliplatin-related thrombocytopenia in patients with gastrointestinal malignancies: a retrospective study
Source: PeerJ. 2023 Oct 13;11:e16230. doi: 10.7717/peerj.16230 (PMC10578303; doi:10.7717/peerj.16230)

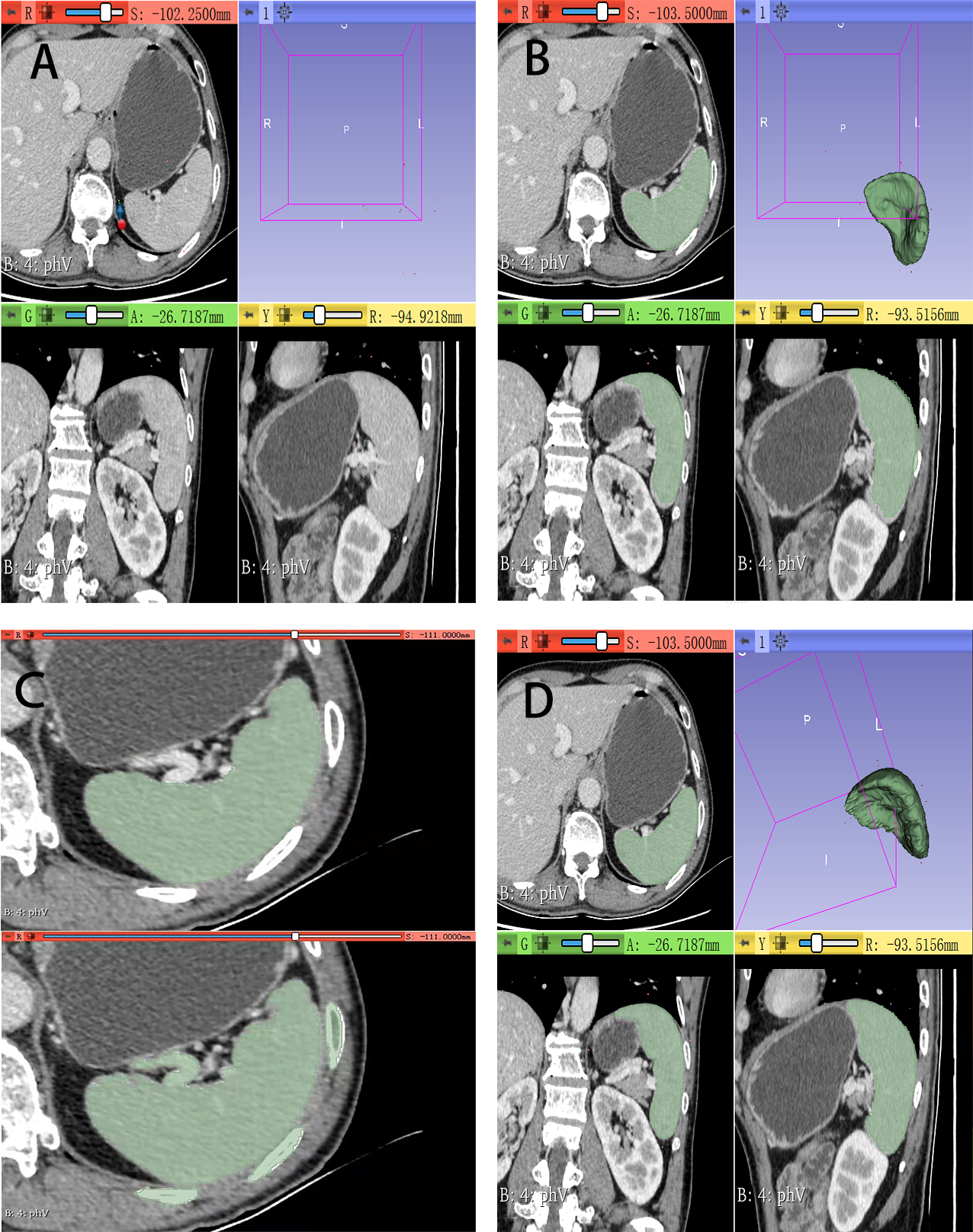

Supplement: Supplemental Information 1 — (A) Boundary points were set near the edge of spleen on axial, sagittal and coronal views. (B) Semi-automatic segmentation of 3D Slicer software. (C) Interactive correction of the segmentation if necessary. Large blood vessels were carefully excluded. (D) The final segmentation. [file peerj-11-16230-s001.png]
