# Supplementary material for: A contrast-enhanced CT-based whole-spleen radiomics signature for early prediction of oxaliplatin-related thrombocytopenia in patients with gastrointestinal malignancies: a retrospective study
Source: PeerJ. 2023 Oct 13;11:e16230. doi: 10.7717/peerj.16230 (PMC10578303; doi:10.7717/peerj.16230)
Supplement: Supplemental Information 2 [file peerj-11-16230-s002.docx]

**Supplementary Table 1.** Oxaliplatin-based chemotherapy regimens.

| Regimens |  |
| --- | --- |
| CAPOX | oxaliplatin 130mg/m^2^ on D1 and capecitabine 1000mg/m^2^ twice a day orally on D1 and D14, repeated every 3 weeks |
| mFOLFOX6 | oxaliplatin 85mg/m^2^ on D1, leucovorin 200mg/m2 on D1 and a bolus of 5-FU 400mg/m^2^ followed by a 46-hour infusion of 5-FU 2.4g/m2, repeated every 2 weeks |
| SOX | oxaliplatin 130mg/m^2^ on D1 and S-1 40mg/m^2^ twice a day orally, repeated every 3 weeks |
