# Supplementary material for: A contrast-enhanced CT-based whole-spleen radiomics signature for early prediction of oxaliplatin-related thrombocytopenia in patients with gastrointestinal malignancies: a retrospective study
Source: PeerJ. 2023 Oct 13;11:e16230. doi: 10.7717/peerj.16230 (PMC10578303; doi:10.7717/peerj.16230)
Supplement: Supplemental Information 3 [file peerj-11-16230-s003.docx]

**Supplementary Data**

Rad-score calculation formula:

Rad-score=-1.0898+0.2033*wavelet-LHL_firstorder_Energy+0.2562*wavelet-HHL_glcm_Idn+0.0893*wavelet-HHL_glszm_HighGrayLevelZoneEmphasis+0.05246*wavelet-LLL_firstorder_Kurtosis+0.0583*wavelet-LLL_ngtdm_Complexity
